# Brain Extraction Using Label Propagation and Group Agreement: Pincram

**DOI:** 10.1371/journal.pone.0129211

**Published:** 2015-07-10

**Authors:** Rolf A. Heckemann, Christian Ledig, Katherine R. Gray, Paul Aljabar, Daniel Rueckert, Joseph V. Hajnal, Alexander Hammers

**Affiliations:** 1 MedTech West at Sahlgrenska University Hospital, Gothenburg, Sweden; 2 Institute of Neuroscience and Physiology, Gothenburg University, Gothenburg, Sweden; 3 Centre for Brain Sciences, Imperial College, London, United Kingdom; 4 The Neurodis Foundation, Lyon, France; 5 Department of Computing, Imperial College, London, United Kingdom; 6 Imaging Sciences and Biomedical Engineering, King’s College, London, United Kingdom; Nanjing University of Aeronautic and Astronautics, CHINA

## Abstract

Accurately delineating the brain on magnetic resonance (MR) images of the head is a prerequisite for many neuroimaging methods. Most existing methods exhibit disadvantages in that they are laborious, yield inconsistent results, and/or require training data to closely match the data to be processed. Here, we present *pincram*, an automatic, versatile method for accurately labelling the adult brain on T1-weighted 3D MR head images. The method uses an iterative refinement approach to propagate labels from multiple atlases to a given target image using image registration. At each refinement level, a consensus label is generated. At the subsequent level, the search for the brain boundary is constrained to the neighbourhood of the boundary of this consensus label. The method achieves high accuracy (Jaccard coefficient > 0.95 on typical data, corresponding to a Dice similarity coefficient of > 0.97) and performs better than many state-of-the-art methods as evidenced by independent evaluation on the Segmentation Validation Engine. Via a novel self-monitoring feature, the program generates the "success index," a scalar metadatum indicative of the accuracy of the output label. Pincram is available as open source software.

## Introduction

A prerequisite for many analytic approaches applied to magnetic resonance (MR) images of living subjects is the identification of the target organ on the image. When images are analysed visually, this usually happens implicitly, ie. the observer’s attention is naturally focussed on the region of interest. Due to the exquisite pattern recognition capabilities of the human visual system, no specific treatment of the image is usually required to achieve this analytic separation. Contrariwise, automatic image analysis generally demands a mask that distinguishes the organ or region of interest from parts of the image that correspond to extraneous structures or to background. For structural 3D imaging of the human brain, especially MR imaging, a variety of brain extraction, skull stripping, or intracranial masking methods have been proposed. These can be distinguished by the level of expert involvement:

1. Manual delineation protocols require a rater to outline the region of interest on all or a subset of the acquired sections [1]. The amount of laborious expert input increases linearly with the number of subjects in the target cohort. Manual delineation results often serve as a gold-standard reference to evaluate new approaches.
2. Semi-automated masking procedures achieve good results at reduced cost, but still require detailed expert interaction with each target image (e.g. MIDAS [2]).
3. Methods that use heuristics to estimate a brain label from a combination of geometric image properties and signal intensities (e.g. Exbrain [3]; FSL BET [4]; SPM [5]) are technically suitable for batch automation with multiple target images. The same is true for approaches that transform a reference label from a standard space into the target space, using the inverse of the normalizing transformation [6].
4. Some algorithms achieve brain labelling on cohorts of images using expert segmentations of a subset of the cohort (e.g. [7–9]). The labelled subset is called atlas, training, or library set. These methods rely on the target images being sufficiently similar to the training set for the algorithm to transfer the expert knowledge. They often require extensive preprocessing, including spatial and intensity normalization.

Users of the automatic methods of categories 3. and 4. should generally apply caution and review each resulting label, because the performance on a given target can vary unpredictably, for example when subtle image artifacts are present. To our knowledge, none of the existing methods provide explicit warnings or performance indices to indicate possible segmentation failures.

In this work, we present *pincram*, a method that fits with those in Category 4) above in that it uses expert brain labels in an atlas set to identify the brain on target images. Development goals were accuracy, robustness, and versatility, and practicality, in particular the ability to process any given target image using atlas data of arbitrary provenance. We also aimed to introduce self-monitoring, ie. the capability of predicting the accuracy of an individual segmentation and warning the user of potentially inaccurate results. Using a variety of sample data sets and experiments, we quantify the extent to which these goals have been attained. Pincram is publicly available at http://soundray.org/pincram as free software under the MIT (“Expat”) License.

## Materials and Method

### Material

**Fig 1:**
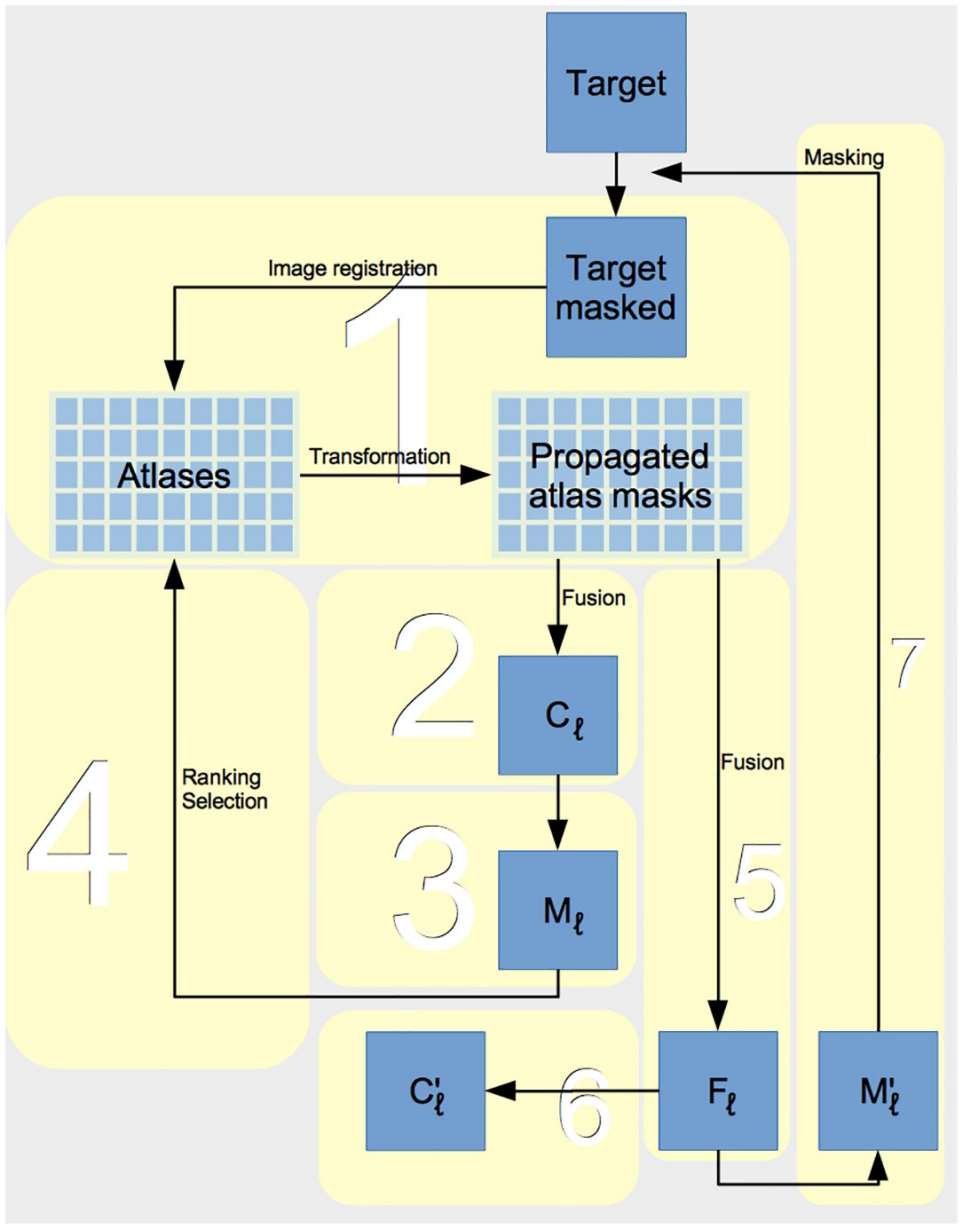
Sample images chosen randomly from each dataset. Images were visually centred at the level of the commissures approximately in the centre of the left thalamus to acquire a transverse (top rows) and a sagittal (bottom rows) slice. Left column, *O, H, L*: manual reference masks, *X*: generated mask (*OX* setup). Middle column, *O, H, L, X*: generated masks (*HX* setup in the case of X). Right column, *O, H, L, X*: discrepancies between the masks—green indicates overinclusion, red indicates underinclusion. Individual JCs were 0.9512 (*O*), 0.9704 (*H*), 0.9647 (*L*), and 0.9503 (*X*).

We used T1-weighted MR images of the human brain obtained from a variety of sources. Scanner manufacturers, sequence characteristics, acquisition strategies, postprocessing, and field strengths varied between the test sets, as well as the visual appearance of the images (Fig 1). We refer to the combination of an image with a spatially corresponding, manually or automatically generated label volume by the term *atlas*.

#### Hammers brain atlases

The Hammers atlases are a publicly available database of manually labelled cranial images of 30 adult subjects (age range 20 to 54, mean 31 years; www.brain-development.org). The images originate from a Signa Echospeed scanner (field strength 1.5 Tesla; GE Medical Systems, Milwaukee, USA) at the National Society for Epilepsy in Chalfont, UK. Further acquisition details are available in Hammers et al. [10]. Image postprocessing consisted in bias field correction (N3 [11]), reorientation along the anterior and posterior commissures, and isotropic reconstruction (0.9375 × 0.9375 × 0.9375 mm^3^) using a single windowed sinc interpolation.

The Hammers atlas database includes spatially corresponding label volumes that identify 83 cortical and subcortical regions. To obtain reference brain masks, we merged these multi-region label sets into a single binary mask. We applied additional processing to fill intracranial regions that are not covered by the resulting label: blurring with a Gaussian kernel (8 mm width), thresholding at 50%, erosion with a 3 × 3 × 3 voxel kernel in two iterations, and merging with the original combined label.

We use *H* as a shorthand reference to the Hammers atlases.

#### OASIS brain atlases

This data set consists of images from 30 healthy subjects (age range 18 to 90, mean 34.3 years), originating from the Open Access Series of Imaging Studies (OASIS) (www.oasis-brains.org/), with labels provided by Neuromorphometrics, Inc. (neuromorphometrics.com/) under academic subscription. The images had been acquired at 1.5 Tesla. The atlases had been published for the *MICCAI 2012 Grand Challenge and Workshop on Multi-Atlas Labeling* [12]. From the original set of 35 atlases, we excluded those that were based on repeat scans of the same subjects. We applied N4ITK bias field correction without masking to the images [13].

The label volumes map 138 regions. As in the case of the Hammers atlases, the labels do not cover the entire intracranial space. We generated brain masks using the filling procedure described in Section *Hammers brain atlases*.

The resulting data set is denoted by *O*.

#### IXI brain images

To obtain images acquired at 3 Tesla, we accessed the publicly available IXI repository of MR images (brain-development.org). The database consists of 575 images of healthy volunteers, 185 of which had been acquired at 3 Tesla at Hammersmith Hospital, London, UK (Intera scanner; Philips Medical Systems, Best, The Netherlands). No segmentation labels were available for this database. We randomly selected 30/185 images and labelled this set as *X* (age range 24.3 to 74.6, mean 49.4 years). From the remainder of 155, we selected another random set of 30 and combined the resulting set with *X* to yield a set of 60, denoted by *X+* (age range 20.2 to 74.6, mean 45.3 years). All images underwent bias field correction with N4ITK without masking.

#### LPBA40 atlases

The LPBA40 repository holds images of 40 adults (age range 16 to 40, mean 29.2 years) imaged at 1.5 Tesla (shattuck.bmap.ucla.edu/resources/ lpba40/ [14]). We obtained the full MR images in native space as well as their skull-stripped versions. We thresholded the skull-stripped images at zero to obtain binary brain masks. We use *L* to refer to this data set.

### Preprocessing

The number of atlases in each of *H*, *O*, *X*, and *L* was doubled by mirroring (left/right flipping) the image and corresponding mask. *X+* was the only data set that was not doubled in this fashion, so it could serve as a reference for evaluating the effect on accuracy of the mirroring procedure.

### Iterative brain labelling procedure

**Fig 2:**
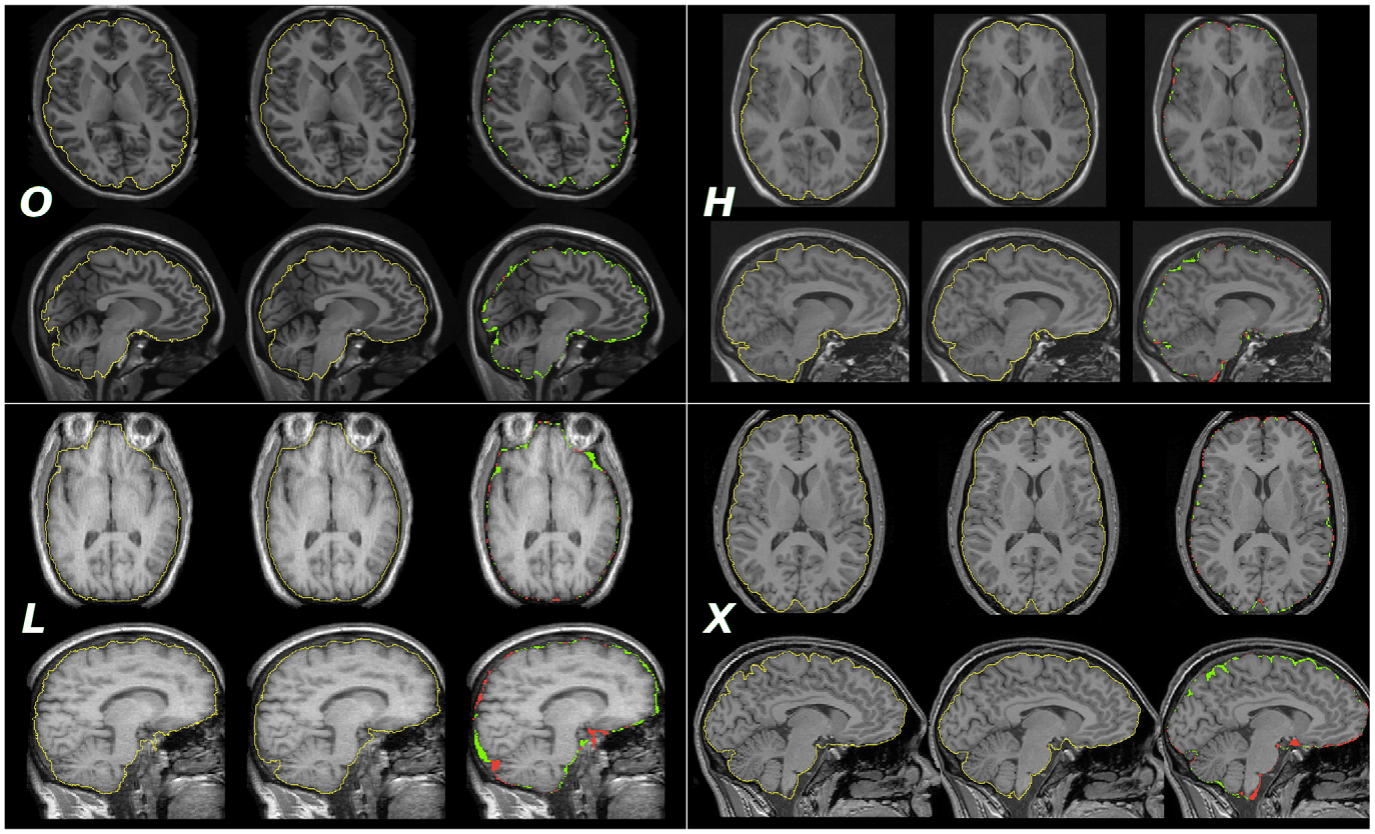
Overview diagram of pincram workflow. Step numbers in the text correspond to numbered boxes. *C*_*l*_: pre-selection fused mask; *M*_*l*_: tight margin (boundary neighborhood) mask; *F*_*l*_: fuzzy label summed from rank-selected subset; $C_{l}^{\prime }$: brain mask generated from *F*_*l*_ by thresholding and binarization; $M_{l}^{\prime }$: wide margin mask generated from *F*_*l*_ by thresholding and binarization

For a given target, labels were generated at three levels of progressive refinement, termed rigid, affine, and nonrigid, according to the type of image registration performed. At each level, a consensus mask was generated. To select a subset of the most suitable atlases to be used at the subsequent level, the similarity of each atlas with the target within the expected boundary region was measured. The expected boundary region was determined by generating a tight mask of the boundary neighborhood (margin) from the consensus mask. A new consensus mask was generated from the selected atlases, and this was used to estimate a more generous margin space to constrain the search in the subsequent level. Fig 2 shows the workflow. A detailed description of the calculations at each iteration or refinement level (*l* ∈ 0, 1, 2) follows. Initially (*l* = 0), the full available set of *n*_0_ atlases was used.

1. Label sets were generated from *n*_*l*_ atlases using a standard label propagation approach based on image registration (cf. Section *Image registration*).
2. The *n*_*l*_ label sets were fused in the target space using summation, thresholding (*t*_*l*_), and binarization, generating a crisp fused brain mask (*C*_*l*_). The value of *t*_*l*_ is configurable and determines the balance between false positive and false negative voxels. A *t*_*l*_ of 50% would correspond to majority voting, but tends to lead to an overly inclusive mask. We found settings of *t*_0_ = 56, *t*_1_ = 60, and *t*_2_ = 60 to yield optimal results.
3. A margin mask *M*_*l*_ was generated, consisting of all voxels within a distance of 4 voxels from the boundary of *C*_*l*_. This serves to reduce the data to be considered in the subsequent ranking evaluation.
4. The *n*_*l*_ individual atlas subjects were ranked in descending order of the similarity (normalized mutual information) of the transformed atlas image with the target image within the margin mask. The *n*_*l*+1_ top-ranking atlases were identified for use in the subsequent level *l*+1, with $n_{l+1}=\max\left\{n_{l}\sqrt[{3}]{8/n_{0}},7\right\}$. This approach ensures that selection only takes place if the number of atlas sets is larger or equal to 10, and that at least 7 atlas sets are used at every level. It also entails more aggressive selection when atlas set sizes are larger.
5. The selected transformed labels were fused by summation to create a fuzzy label *F*_*l*_.
6. *F*_*l*_ was thresholded (*t*_*l*_) and binarized in the target space, generating a second crisp fused brain mask $C_{l}^{\prime }$.
7. *F*_*l*_ was converted to a crisp margin mask by setting all voxels with values between 15% and 99% to 1, and all other voxels to zero. The resulting margin mask $M_{l}^{\prime }$ was applied to the target used to restrict the region within which registration parameters were optimized in the subsequent level. This ensured that only the margin that presumably contained the true boundary was considered for the similarity calculation during the registration step in the subsequent iteration.

The final fused brain mask $C_{2}^{\prime }$ was retained as the result label. *C*_0_ and *C*_1_ were discarded except for an experiment that demonstrates the evolution of the brain mask through the three iterations (cf. Section “Effect of registration level on accuracy”).

### Image registration

Image registration consisted in optimizing a transformation based on maximizing normalized mutual information between a given atlas and target [15]. Initially, the degrees of freedom were set to 6 (rigid translation and rotation). For affine registration, scaling and shearing were additionally allowed (12 degrees of freedom). Nonrigid registration consisted of applying displacements to the atlas image via a lattice of control points (spacing 6 mm), blended using B-spline basis functions. The stopping condition for the optimization was either no further improvement in similarity or reaching 40 iterations.

All registration steps were carried out using the Image Registration Toolkit (IRTK, https://github.com/BioMedIA/IRTK).

### Experiments

#### Notation and overview

We use the notation *AB* to refer to an experiment where a data set *A* consisting of multiple atlases was used to segment images from set *B*. The source of reference data for evaluating the segmentation result is denoted by *:C*. Thus, *AB:B* denotes an experiment where *A* was used to segment *B*, and the result was evaluated with the reference brain masks available in *B*. Since the atlas generation can be chained, an arbitrary number of letters can appear before the colon. For example, *ABCC:C* would denote an experiment where *A* was used to segment *B*; the resulting segmentations were used to build a new atlas set, which was applied to C; the result was again used to build an atlas set, which was applied to C again, and finally the segmentations *ABCC* were compared with the reference segmentations in set *C*.

An overview of the experiments is shown in Table 1.

**Table 1:** Overview of experiments.

Experiment	Setup	Characteristic to be assessed
HH:H	Leave-two-out cross-comparison	Within-group accuracy
OH:H	Cross-scanner (same FS)	Robustness towards scanner diff.
OXH:H	Cross-scanner (different FS)	Robustness towards FS diff.
OXHH:H	Cross-scanner with customization	Effect of customization
OX+H:H	Cross-scanner without mirroring	Effect of mirroring
OO:O	Leave-two-out cross-comparison	Within-group accuracy
HO:O	Cross-scanner (same FS)	Robustness towards scanner diff.
HXO:O	Cross-scanner (different FS)	Accuracy across FS
HXOO:O	Cross-scanner with customization	Effect of customization
HX+O:O	Cross-scanner without mirroring	Effect of mirroring
LL:L	Leave-two-out cross-comparison	Within-group accuracy
HL:L	Cross-scanner	Robustness towards scanner diff.
HL30L:L	Cross-scanner with selection	Effect of forward selection
HX:OX	Cross-scanner	Consistency
OX:HX	Cross-scanner	Consistency
X+H:H	Cross-scanner	Influence of varying *n*_0_

FS: field strength, diff.: differences

#### Quality measures

To assess the quality of a generated brain label, we assessed its agreement with the reference mask using four complementary measures: the overlap ratio or Jaccard coefficient (JC; intersection divided by union), the volume error, the sensitivity [intersection divided by (intersection + false negatives)], and the 95th percentile of the symmetric surface distance. We did not determine specificity (true negative rate), because it depends on the size of the background and is therefore meaningless for comparisons between data sets with differing fields of view.

Overlap measures do not yield information about volume differences between the label pair. We therefore determined volume error as a percentage, calculated as

$$\Delta V=200\cdot \frac{V_{r}-V_{g}}{V_{r}+V_{g}}$$ (1)

where *V*_*r*_ is the volume of the reference label and *V*_*g*_ the volume of the generated label.

Since averaging of positive and negative volume errors can hide poor performance, we also calculated the mean of |Δ*V*|.

We determined the JC between the final fused brain mask $C_{2}^{\prime }$ (generated at Step 6, fused from *n*_3_ transformed atlas labels) and mask *C*_2_ (generated at Step 2, fused from *n*_2_ transformed atlas labels). We investigated the value of this “success index” as a predictor of segmentation accuracy that is independent of reference segmentations.

#### Within-group cross-comparisons

To determine within-group performance of pincram, we carried out three leave-two-out cross-comparisons; one within each of the Hammers (*HH:H*), OASIS (*OO:O*), and LPBA40 (*LL:L*) set. Each image was labelled in turn, using *n*−2 data sets as atlases, excluding the target itself as well as the mirrored version of the target.

#### Cross-scanner accuracy

Most atlas-based methods require atlas and target images to have similar intensity characteristics. The mapping between signal and grey scale value varies between scanners to the extent that pairwise registration between images from different sources can fail [16]. In developing pincram, we overcame this restriction thanks to the robustness of the underlying registration algorithm, including the use of normalized mutual information as the optimization target [17]. This section describes a series of experiments we used to assess segmentation accuracy in cross-scanner scenarios.

We segmented the Hammers atlas images using the OASIS atlases, comparing the resulting labels with the Hammers reference masks (*OH:H*). We then inverted the setup and segmented the OASIS images using the Hammers atlases, comparing the results with the OASIS labels (*HO:O*).

Both the Hammers and OASIS images had been acquired at 1.5 T. We could not set up a similarly simple experiment showing segmentation accuracy across field strengths (e.g. *HX:X*), because no reference masks *:X* were available for the 3 T IXI images. We were, however, able to obtain indirect performance measures that can indicate the applicability of pincram across field strengths in the following experiments.

We segmented a set of 30 IXI images twice: once with the Hammers atlases and once with the OASIS atlases. We determined the agreement of the two label sets (*HX:OX*) as a measure of consistency as demonstrated in previous work (cf. Fig 5 in Heckemann et al. [18]).

We subsequently generated two “synthetic” (based on automatic segmentations) atlas sets from the 3 T images and measured the performance of these sets when applied to 1.5 T image data. The Hammers-based synthetic atlas set (*HX*, 30 unflipped and 30 flipped atlases) was used to segment the OASIS images, and the OASIS-based synthetic atlas set (*OX*, 30 unflipped and 30 flipped atlases) was used to segment the Hammers images. Both result sets were then compared to the original manual segmentations (*HXO:O*, *OXH:H*). The accuracy of the output segmentations is thus affected by two traversals of the field-strength boundary between the respective data sets. The accuracy of the direct segmentations (Hammers with OASIS, *OH:H* and vice versa, *HO:O*) served as a reference for estimating the size of the accuracy-reducing effect.

#### Secondary synthetic atlases

We set out to assess the effect on accuracy of customizing an atlas set to an ensemble of target images. The reasoning was that if pincram yields inaccurate results in a part of a given ensemble due to acquisition differences between the atlas and target images, accuracy is potentially recoverable by exploiting the similarity of the images within the ensemble.

We set up three experiments of this type, one using a subset of the target ensemble and two using the full target set to generate synthetic atlases.

To assess the effect of forward selection on accuracy, we used the Hammers atlases to segment the LPBA40 images and evaluated the resulting segmentations against the reference masks provided with the latter (*HL:L*). From the thirty most successful segmentations on the JC criterion, we built an atlas set *HL*_30_, applied this back to all 40 LPBA40 sets, and compared with the reference masks (*HL_30_L:L*).

Since a reference for evaluating segmentation success is not normally available, we examined the effectiveness of non-selectively building a synthetic atlas set from all images in the target ensemble. We used the *HXO* and *OXH* data described in the previous section to this end, segmenting again the data in *O* and *H*, respectively. The shorthand notation is thus *HXOO:O* and *OXHH:H*.

#### Effect of mirroring on atlas accuracy

Adding left/right-flipped versions of each atlas and label is a frequently employed procedure that doubles the size of an atlas resource [7, 8], exploiting the slight shape asymmetry that occurs naturally in the head. Whether an atlas database that has been enlarged in this fashion is equivalent to an equally large database consisting only of original, native images is open to question. To find the answer in the context of using pincram, we compared the *OX* and *HX* sets described above (Section *Cross-scanner accuracy*), which had been doubled by mirroring, with sets consisting of 60 native images each (*OX+* and *HX+*). Again, we used the OASIS-derived atlases to segment Hammers images and the Hammers-derived atlases to segment OASIS images. We compared the results of the experiment *OXH:H* with those of *OX+H:H*. Likewise, we compared *HXO:O* with *HX+O:O*. We assumed that significantly lower quality measures for the doubled sets would indicate that the doubling manoeuvre incurs a cost in terms of accuracy.

#### Effect of varying atlas number

For all experiments described above, we set the number of atlases *n*_0_ to the maximum possible, given the size of the database and the experimental constraints. To examine the impact on accuracy of using fewer than the available atlases, we set up the following experiment. We chose *X+* as the atlas set, with labels generated from H. This enabled a maximum *n*_0_ of 60. As the target set, we chose *H* (*n* = 30). We then obtained random subsets of varying size (*HX+_*n*_H:H* with *n* ∈ 7, 9, 12, 17, 25, 38, 60) and used these to segment *H*, yielding runs of the pattern *HX+_*n*_H:H*.

#### Effect of registration level on accuracy

For one of the experimental runs described in the previous section, *HX+_60_H:H*, we retained the masks $C_{0}^{\prime }$ (rigid level output, generated from 30 selected atlases (*n*_1_)) and $C_{1}^{\prime }$ (affine level output, *n*_2_ = 15), in addition to the final label $C_{2}^{\prime }$ (nonrigid level output, *n*_3_ = 7). For each mask, we determined JC with the reference masks, enabling comparison to assess the impact that the registration refinement had.

#### Comparison with FSL BET

The brain extraction tool (BET, [4]) from the FSL suite is widely used. We therefore applied BET to the *H* and *O* cohorts to provide an additional point of reference for assessing the results we obtained with pincram. We used the invocation “bet image mask -R -m”. The parameter “-R” improves the tool’s results by iterating the brain extraction to achieve a robust brain centre estimation. The parameter “-m” is required for saving the binary output mask. For each subject, we assessed the overlap of the output mask with the reference mask using JC.

#### Computing environment

The experiments were carried out in a mixed cluster environment at the Centre for High Performance Computing at Imperial College, London. We recorded the runtime and resource allocation for the pincram procedure in one experiment, *HX+O:O*. The 30 target images were segmented on cluster servers equipped with 16 GB RAM and two quadruple-core Xeon E5-2620 CPUs (Intel Corp., Santa Clara, CA, USA) clocked at 2.0 GHz. The task was configured so that it ran sequentially, ie. all atlas-target registrations for a given target ran in succession on a single core. This allowed a more meaningful assessment of the runtime behaviour than a parallelized configuration where different registration subtasks are processed on diverse server hardware.

### Independent evaluation

We followed the procedure for independent evaluation of brain extraction methods provided by the Segmentation Validation Engine (SVE, sve.bmap.ucla.edu [19]). The program we used for the submission was an early development version of pincram. The current version could not be evaluated because the SVE was closed to new submissions at the time of manuscript preparation.

## Results

**Fig 3:**
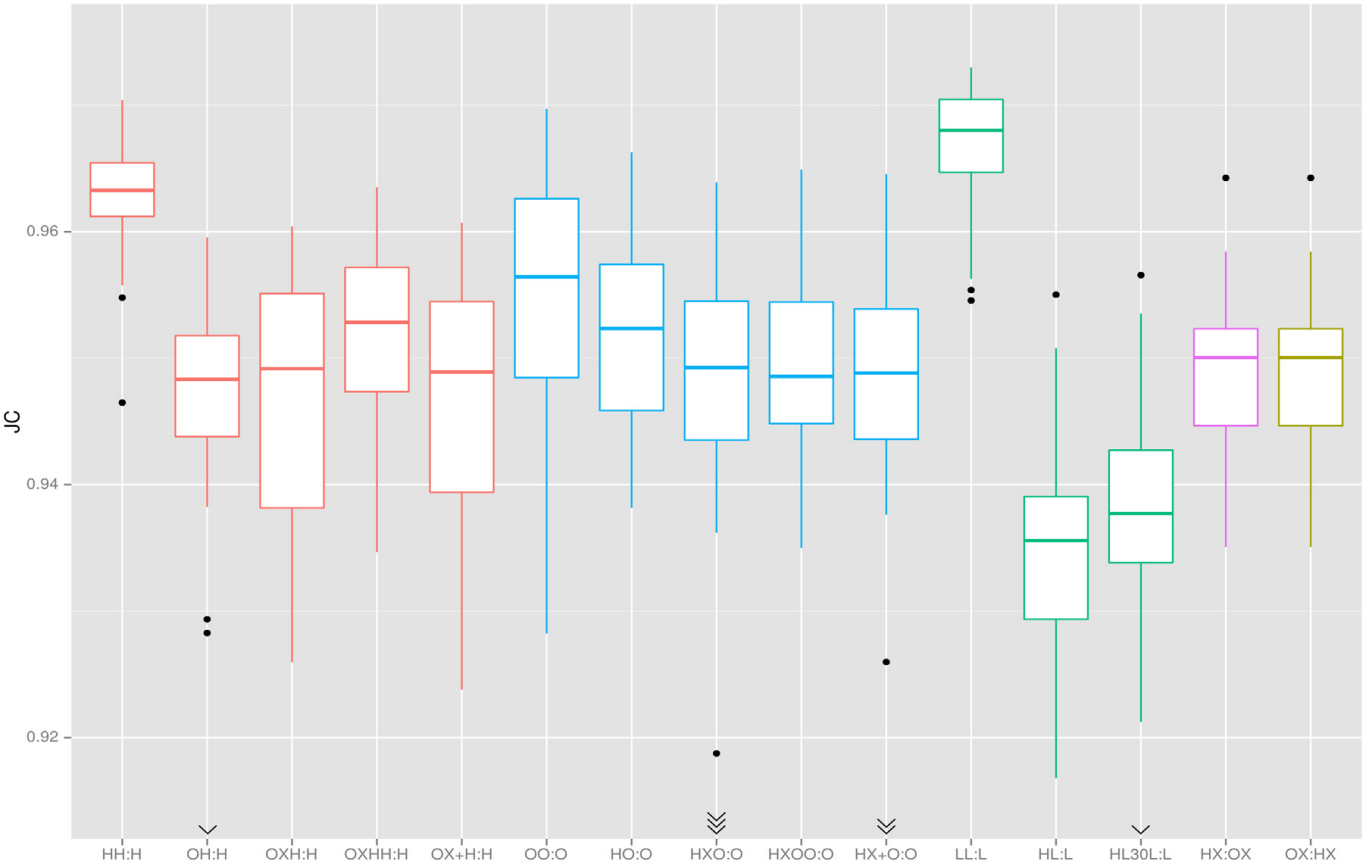
Jaccard coefficients. Colours distinguish target data sets. Centre lines: median, boxes: interquartile range, whiskers: truncated range, dots: outliers, arrowheads: off-scale outliers.

**Fig 4:**
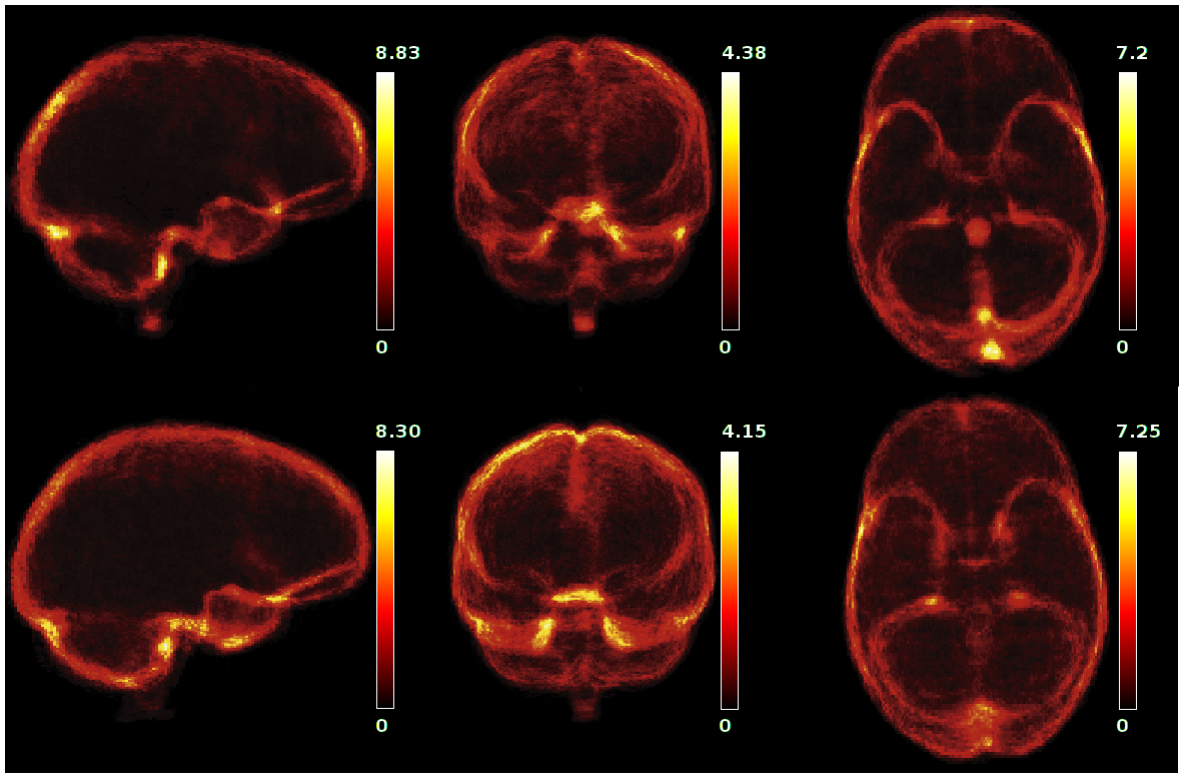
Projection maps for *LL:L*. Binary error maps images identifying false negative and false positive voxels were generated for all 40 individuals. After spatial normalization to a subject with a typical head shape (Subject 32) and averaging of the error maps, projection maps were created by summation along the cardinal axes. The maps are scaled individually to maximize the dynamic range. The procedure was adapted from http://sve.bmap.ucla.edu/instructions/metrics/projections/. Top row: false positive, bottom row: false negative.

Table 2 and Fig 3 summarize all experimental results by target data set. The corresponding raw data are provided as a supplement (S1 File). Fig 4 shows projection maps highlighting the predominant locations of false positive (top row) and false negative (bottom row) errors for experiment *LL:L*. The brain volume as estimated by pincram was 1.37 L on average, with a typical coefficient of variation of 10.3%. The volume of the reference masks was 1.35 L on average, with similar variation (9.7%). Volume error was positive in the majority of individual setups.

**Table 2:** Volumes and accuracy.

Pairing	*n*_0_	Tgt (n)	Ref Litre	CV %	Gen Litre	CV %	Δ*V* %	|Δ*V*| %	Overlap JC	Overlap Dice	Sensitivity *mm*	SD95
HH:H	29×2	30	1.343	10.17	1.354	10.15	0.870±0.930	1.069±0.683	0.9628±0.0049	0.9810±0.0025	0.9854±0.0049	1.857±0.462
OH:H	30×2	30	1.343	10.17	1.377	10.48	2.495±1.847	2.495±1.847	0.9458±0.0107	0.9721±0.0057	0.9845±0.0052	2.975±0.958
OXH:H	30×2	30	1.343	10.17	1.367	10.55	1.737±1.882	1.868±1.748	0.9463±0.0105	0.9724±0.0055	0.9810±0.0055	3.020±0.948
OXHH:H	30×2	30	1.343	10.17	1.365	10.58	1.649±1.234	1.697±1.165	0.9522±0.0076	0.9755±0.0040	0.9836±0.0048	2.712±0.743
OX+H:H	60	30	1.343	10.17	1.366	10.66	1.647±1.770	1.765±1.649	0.9469±0.0100	0.9727±0.0053	0.9808±0.0056	2.968±0.856
OO:O	29×2	30	1.333	10.11	1.368	10.21	2.617±1.677	2.707±1.521	0.9548±0.0106	0.9768±0.0056	0.9898±0.0052	2.101±0.502
HO:O	30×2	30	1.333	10.11	1.362	10.12	2.189±1.291	2.224±1.228	0.9519±0.0076	0.9753±0.0040	0.9862±0.0060	2.562±0.363
HXO:O	30×2	30	1.333	10.11	1.419	10.87	6.182±10.572	6.182±10.572	0.9237±0.0797	0.9583±0.0497	0.9891±0.0055	3.694±3.159
HXOO:O	30×2	30	1.333	10.11	1.374	10.04	3.093±1.303	3.093±1.303	0.9493±0.0077	0.9740±0.0040	0.9893±0.0053	2.672±0.384
HX+O:O	60	30	1.333	10.11	1.405	11.22	5.135±9.387	5.135±9.387	0.9315±0.0703	0.9630±0.0441	0.9885±0.0055	3.351±2.781
LL:L	39×2	40	1.358	9.42	1.356	10.57	-0.166±1.178	0.925±0.733	0.9666±0.0047	0.9830±0.0024	0.9822±0.0050	1.748±0.213
HL:L	30×2	40	1.358	9.42	1.282	10.40	-5.734±1.219	5.734±1.219	0.9349±0.0079	0.9663±0.0042	0.9395±0.0095	2.850±0.360
HL_30_L:L	30×2	40	1.358	9.42	1.288	10.52	-5.310±1.218	5.310±1.218	0.9377±0.0080	0.9678±0.0043	0.9428±0.0094	2.691±0.358
HX:OX	30×2	30	1.373	8.12	1.400	8.24	1.970±1.183	1.999±1.132	0.9489±0.0062	0.9738±0.0033	0.9835±0.0046	3.213±0.514
OX:HX	30×2	30	1.400	8.24	1.373	8.12	-1.970±1.183	1.999±1.132	0.9489±0.0062	0.9738±0.0033	0.9643±0.0081	3.213±0.514

Atl: atlases, Tgt: targets, Ref: Reference, CV: coefficient of variation, Gen: generated, Δ*V*: Volume error, |Δ*V*|: absolute volume error, SD: standard deviation, JC: Jaccard coefficient, Dice: Dice coefficient, SD95: symmetric surface distance (95th percentile). ± indicates standard deviation

As expected, the overlaps were largest (JC > 0.96) for the within-group comparisons, with *LL:L* leading the ranking. This may be due to the strong similarity between the images in *L*. Also, the reference masks appeared visually smoother and more generous for *L* than for *H* and *O*. The mean volume of the reference masks was larger in *L* than in the other two, albeit not significantly so (1.36 L (*L*); 1.34 L (*H*), 1.33 L (*O*)). The variation of overlaps in *HH:H* and *LL:L* was small, whereas for *O* it was twice as high, indicating that the reference segmentations may not have been as consistent in *O* as in the other data sets.

In cross-scanner setups, overlaps are only slightly reduced. For example, while the *HH:H* setup yielded an overlap of 0.963± 0.005, the *OH:H* setup resulted in a mean JC of 0.946± 0.011, with a single outlier at 0.905. (The segmentation quality of the outlier would still be acceptable for many applications.) Some of the discrepancy may be due to limitations of cross-scanner accuracy, but also to differences between *H* and *O* in terms of the manual segmentation protocols. We note that the generation of the *OXH* atlas involved crossing the field strength boundary twice, ie. we segmented a 3 T data set (*X*) with a 1.5 T atlas set (*O*) and used the resulting set of synthetic atlases to segment a different 1.5 T set (*H*). The fact that the *OXH:H* setup yields approximately the same overlap as *OH:H* while eliminating a low outlier suggests that the agreement-reducing effect of scanner differences is small by contrast with the protocol difference. Going one step further and generating a “custom” atlas for *H* on the same basis even yields a significant improvement (*OXH:H*: 0.946, *OXHH:H*: 0.952, p < 10^−6^). The setup where half of the atlas images are mirrored (*OXH:H*) yields the same overlap results to three decimal places as the one where the atlas consists of only native images (*OX+H:H*).

**Fig 5:**
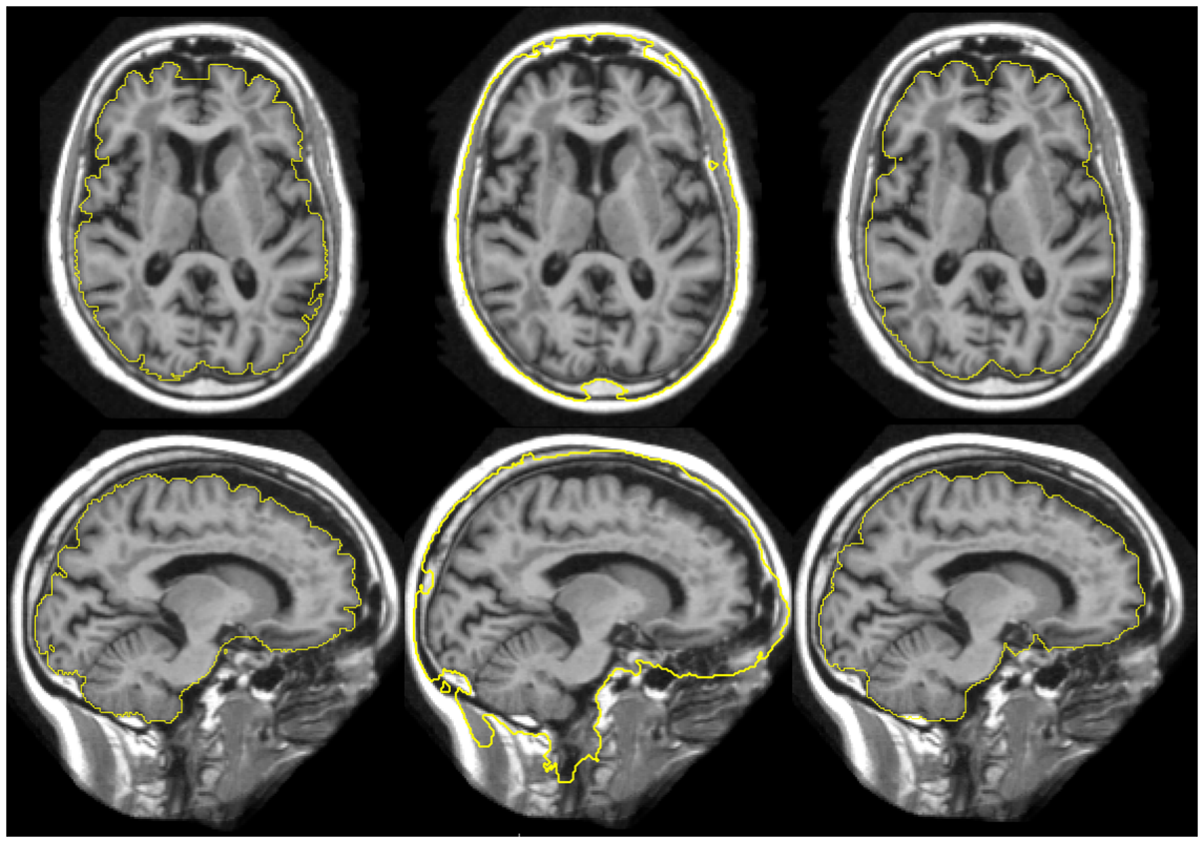
Subject 30 of **O**. Left: with overlay of reference segmentation. Centre: with overlay of failed generated segmentation (*HXO:O*) (one of four outliers in Fig 3; JC 0.592, success index 0.884). Right: with overlay of successful segmentation using customized atlas (*HXOO:O*, JC 0.937, success index 0.977)

The five segmentation experiments carried out on *O* targets corroborate the observations made on *H* targets. We note in addition that the *HXO:O* setup produced four outliers. On visual review, the relevant images were noticeably different from the remainder of the *O* cohort in that they showed pronounced degrees of atrophy and white-matter disease, as well as an unusual intensity distribution where subcutaneous scalp fatty tissue appears substantially brighter than grey and white brain matter. Other images in set *O* presented this intensity pattern to a lesser degree. The “custom” atlas setup *HXOO:O* did not lead to an improvement of the mean overlap, but it eliminated the outliers and produced visually similar segmentations for all target images (cf. example in Fig 5). The setup that replaced mirrored images with native images in the atlas set (*HX+O:O*) yielded similar results to the standard setup (*HXO:O*), with one fewer failed segmentation.

The customization experiment where we employed a selection step to include only the “best” secondary atlases also yielded the expected result, although the extent of the improvement of *HL_30_L:L* over *HL:L* was small (0.938 compared to 0.935, p < 10^−8^). Both sets of automatic segmentations (*HL* and *HL_30_L*) were consistently and substantially smaller than the reference segmentations *L* (volume error -5.7% and -5.3%). On visual review, we did not observe problematic underinclusions in the generated masks. A substantial number of discrepancies appear in the hindbrain region, where the reference masks include larger parts of the medulla oblongata than the atlas masks.

The overlap results between *HX* and *OX* indicate strong agreement and high consistency (JC 0.950± 0.0036), showing that pincram yields similar results in spite of the differences between the atlas sets *H* and *O*.

**Fig 6:**
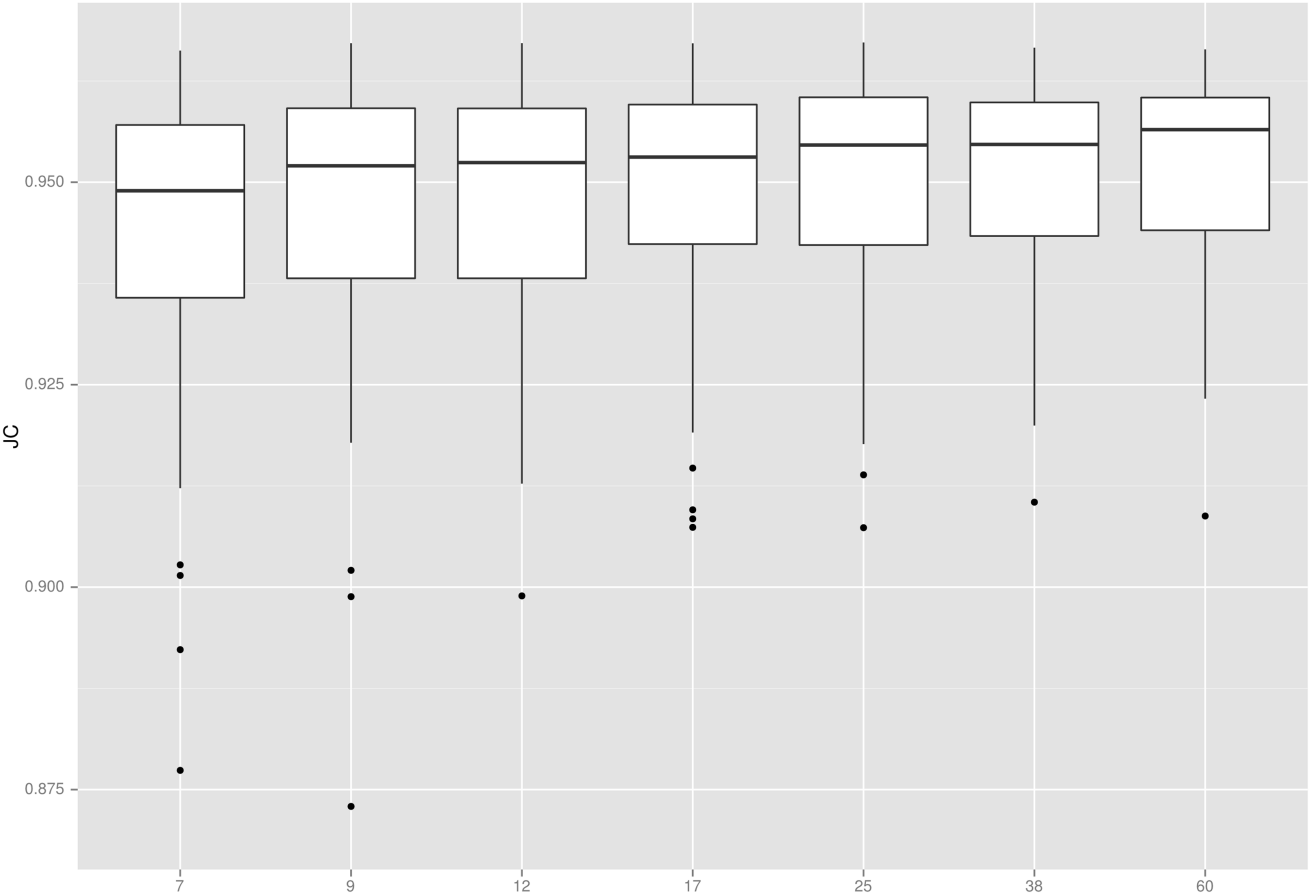
Number of atlases used versus accuracy achieved. X axis: *n*_0_ (ordinal scale), y axis: JC. Boxplot features as in Fig 3.

The strongest agreement between generated and reference segmentations was achieved when using all available atlases. Reducing the number of atlases leads to a reduction in accuracy, as shown in Fig 6. Although significant (*n*_0_ = 7 versus *n*_0_ = 60: *p* = 4 ⋅ 10^−5^ on paired t-test), the difference is small. The spread and number of outliers, however, and the outliers’ relative degree of failure, diminish markedly with increasing numbers of atlases up to 25.

The registration level had a substantial influence on the accuracy of the generated masks. In Experiment *HX+_60_:H*, the mean JC across 30 target subjects was 0.900 ± 0.0262 for $C_{0}^{\prime }$, 0.917 ± 0.0148 for $C_{1}^{\prime }$, and 0.951 ± 0.0140 for $C_{2}^{\prime }$.

A typical segmentation, involving one target brain and 60 atlas brains, occupied a single CPU core for 195± 28 minutes.

**Fig 7:**
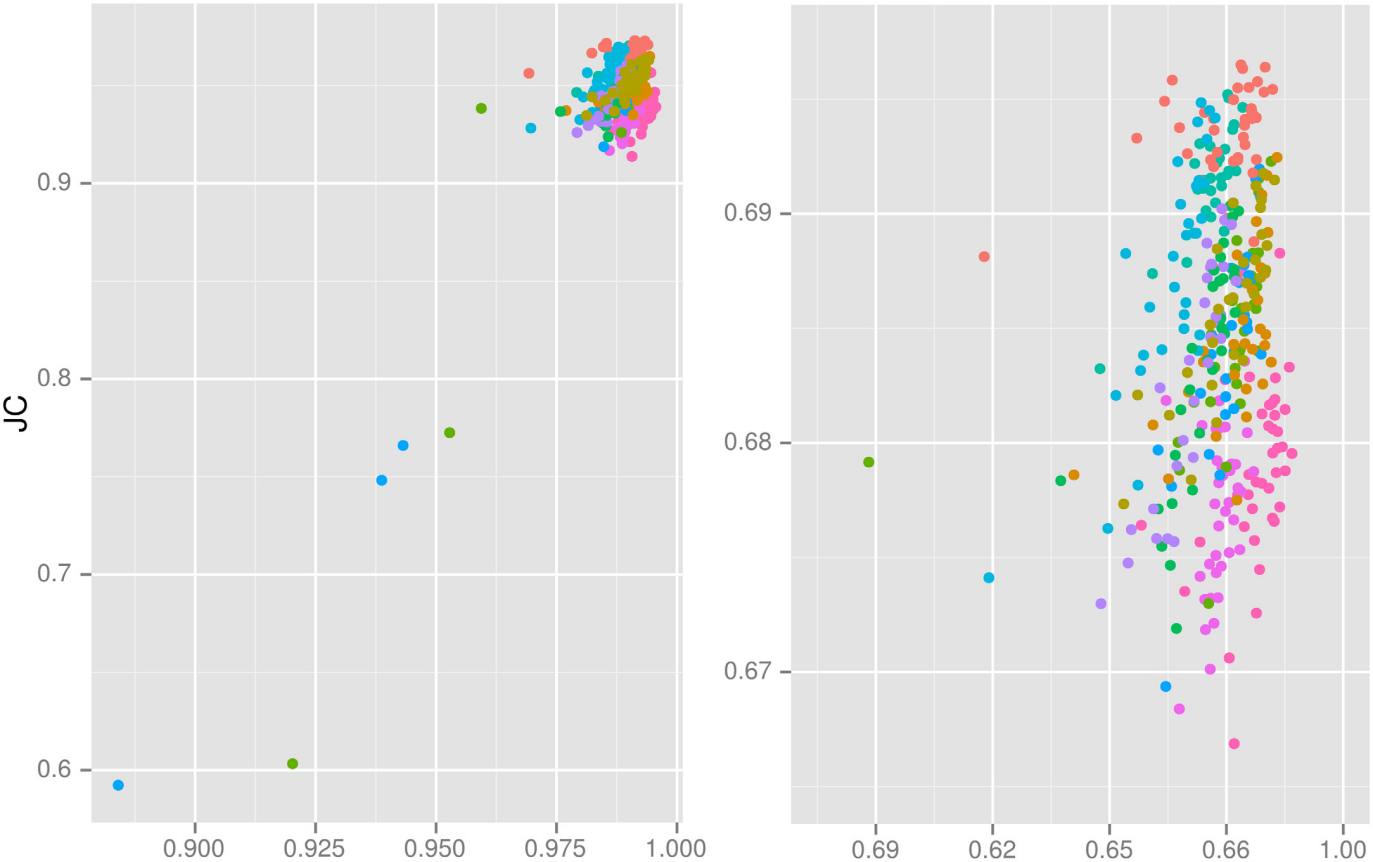
Scatterplot showing success index versus true accuracy. X axes show the success index (JC between $C_{2}^{\prime }$ and *C*_2_), and y axes show JC between the final generated mask $C_{2}^{\prime }$ and the gold-standard reference label. Left: full range. Right: zoomed on non-outlier data (five outliers are off-scale). Experiments (corresponding to rows in Table 3) are grouped by colour.

The success index—the agreement between the final output mask ($C_{2}^{\prime }$) and its predecessor (*C*_2_)—correlated strongly with the agreement of $C_{2}^{\prime }$ with the target reference masks in the experiments where such a reference was available (Table 3, Fig 7). At a cutoff value of 0.96, the index would have drawn attention to all five low outliers in this set of experiments.

**Table 3:** Correlation of the success index with JC.

Pairing	r	p
LL:L	0.35	2.7 ⋅ 10^−2^
HXOO:O	0.62	2.5 ⋅ 10^−4^
OXHH:H	0.81	4.6 ⋅ 10^−08^
HX+O:O	0.93	4.7 ⋅ 10^−14^
OX+H:H	0.73	4.8 ⋅ 10^−06^
HH:H	0.83	1.2 ⋅ 10^−08^
OO:O	0.79	1.6 ⋅ 10^−07^
HXO:O	0.99	*
OXH:H	0.90	2.0 ⋅ 10^−11^
HL:L	0.37	1.7 ⋅ 10^−2^
HL30L:L	0.42	7.0 ⋅ 10^−3^

Pearson’s r and p-value.

*: p-value below smallest representable number

The JC overlap results obtained with FSL BET were lower than those obtained with pincram in all tested cases. For the *H* cohort, the average JC was 0.933 ± 0.010 (compare with “Overlap JC” in Table 2, rows 1 to 5); for *O*, it was 0.810 ± 0.074 (compare with “Overlap JC” in Table 2, rows 6 to 10). Deviations from the reference masks occurred predominantly as overinclusions in the pharynx region.

Evaluation results on the SVE placed pincram on rank 3 of the archive (submission number #287). For the early development version of pincram that we used for the SVE evaluation, the Jaccard coefficient was 0.9631± 0.0067, corresponding to a Dice coefficient of 0.9812± 0.0035. The sensitivity was 0.9883 ± 0.0038 and the specificity 0.9953± 0.0019. At the time of manuscript preparation, the SVE site was closed for new submissions; we could therefore not test the current version. One of the higher-ranking entries is “groundTruth”, an apparent test submission that does not pertain to a competing method (#229). The other higher-ranking entry (#292) was produced with NICE (nonlocal intracranial cavity extraction [9]) and records a Jaccard coefficient of 0.9645 ± 0.0046, a small (within one standard deviation) but significant (p = 0.01) improvement on the pincram prototype.

## Discussion

This study describes a new atlas-based brain masking method, *pincram*, and provides strong evidence of its accuracy and robustness in a series of experiments mimicking real-world brain extraction tasks. The method combines a set of tried and tested approaches in a new fashion (label propagation [20] using nonrigid registration [15], atlas selection [21, 22], and margin-mask based data reduction [8]). It includes a novel self-monitoring feature that produces a success index as a metadatum. The method is implemented as open-source software, available for download from http://soundray.org/pincram under the MIT (“Expat”) licence.

While other library-based methods are similarly [7, 8, 23] or more accurate [9], they tend to have considerable restrictions in practice. Pincram is unique among library-based methods in that it processes any given T1-weighted target head image on the basis of data sets of labelled T1-weighted images acquired on other scanners. Users of pincram will thus have a broad choice of atlas sets, as several are freely available with moderate licence restrictions. Other methods will only work on images acquired with the same sequence on the same type of scanner as the atlas data set. The image registration programs that pincram employs (rreg2, areg2, and nreg2 [15, 24, 25]) are comparatively robust towards differences in image intensity distributions—even those resulting from differences in field strength—thanks to the use of normalized mutual information as the optimization target. The resulting advantage is that pincram does not require laborious generation of a target-specific atlas set to achieve segmentations of reasonable accuracy.

Nevertheless, a custom atlas set can yield benefits of accuracy, even if automatically generated, as exemplified in our experiments *HXOO:O* and *OXHH:H*. Another experiment, *HL_30_L:L*, shows similar benefits when a subset of the target set is turned into the custom atlas set via a selection step. While the advantage over the *HL:L* setup was statistically significant, it was small. A possible explanation for this observation is that the pincram procedure itself detects and applies the most suitable atlases from a given set, so that a small number of unsuitable atlases does not influence the end result negatively. We also observed that low outliers resulting from using the unrelated atlas set were eliminated with the custom atlas, pointing to a further increase in robustness through the customization approach.

The slight variation in the results of the three cross-validation experiments (*HH:H*, *OO:O*, and *LL:L*) can most likely be attributed to differences in the production of these atlases, specifically between segmentation protocols and the consistency with which they have been applied. Set *L* uses a generous definition with smooth mask boundaries. In *H* the boundary follows the gyri more closely, resulting in a larger surface and more opportunities for the automated procedure to label voxels inaccurately. The accuracy is still very high. Both in *H* and *L*, the definitions have been applied consistently between subjects. Variation is higher and accuracy slightly reduced for *O*.

Our results lend tempered support to the practice of flipping or mirroring atlases to double the size of an atlas resource. Both relevant experiments show near-equivalent numeric results for doubled versus same-size native atlas sets. Still, the fact that using the native atlas set resulted in one fewer low outlier means that the mirroring manoeuvre does incur a cost that practitioners should assess on a case-by-case basis.

Our findings suggest that using all available atlases in a data set yields optimal segmentation results. However, the overall impact of reducing the number of input atlases is so small as to be negligible for many practical purposes. Thus, pincram can, within reason, be used with fewer atlases if computation time is a concern. Fig 6 gives an indication of likely effects.

We restricted our investigation to adult brains, leaving an evaluation on children and infants to future work. However, we showed previously that label propagation with our image registration procedure works for subjects down to 2 years of age, even when the atlas set consisted of images of adults [26]. Brains of 1-year olds can be segmented automatically with atlases of 2-year olds [27]. Brains of neonates cannot be segmented automatically with adult atlases, secondary atlases of 2-year olds, or tertiary atlases of 1-year olds. Neonatal brain image segmentation using label propagation works if a manually generated atlas of term-born and preterm infants is available [28, 29].

The development of pincram followed a top-down, rapid-prototyping model to suit the authors’ needs in preparing image repositories of varying size and provenance for morphometric analysis. Parameters were found through trial and error. One of the consequences is the slight bias that the method shows towards overestimating the brain volume, as for many morphometric analyses, the penalty for overinclusions (false positives) is much smaller than for underinclusions. In particular, we took pains to minimize the risk of excluding grey matter at the gyral crowns and tolerated the side effect that small portions of the exterior cerebrospinal fluid spaces or meninges can occasionally be included in the generated brain masks. For brain extraction tasks that predetermine other priorities, users may need to modify configuration settings, in particular those that influence the extent of the margin masks.

We restricted our investigation to the task of producing a brain mask that includes the ventricular and sulcal fluid spaces. With suitable atlas sets, pincram could in principle be used to produce full intracranial masks. The difference between the two is small for young or middle-aged healthy adults, but in ageing and dementia, brain atrophy leads to a reduced volume of the former type of mask, while masks of the intracranial space should remain unaffected. Similarly, we could have addressed the problem of generating soft-tissue brain masks (grey and white matter, excluding ventricles). We chose to leave these challenges to future work. We focussed instead on the most common type of brain mask, which enabled us to use several atlas sets from diverse sources and to thoroughly investigate the accuracy and robustness of our method in realistic scenarios.

Independent evaluation of a prototype pincram version on the SVE shows a level of accuracy that to date remains superior to all competing methods, with the exception of NICE by Manjon et al. [9]. We note, however, that the differences between the top-ranked methods are very small (in the third decimal place of the overlap measures) and thus not likely to be relevant in practice. Improvements on pincram since this early version have focussed on robustness and practicality, rather than accuracy. We would thus expect the current version to yield a similar SVE result, if and when SVE becomes available again. Among the five top-rated brain extraction methods on SVE, pincram is the only one that enables users to segment their own images with a publicly available atlas set.

When carried out in sequence, the calculations involved in applying pincram to a given target are fairly time-consuming. This can be mitigated by parallelization. The current version makes a provision for shell-level parallelization of the registration subtasks via the ‘-par’ option. The most computationally intensive parts of the calculation can thus be distributed to multiple CPU cores. In practice, we found that this reduces calculation times from 195 to circa 15 minutes. Further optimization of the code will lead to quicker turnaround times for single subjects. However, optimal throughput for larger target ensembles is achieved using the sequential task configuration (option ‘-par 1’) and processing as many targets in parallel as the given architecture allows.

Pincram logs information that enables users to predict runtime behaviour and, more importantly, segmentation quality. In homogeneous data sets processed with a suitable atlas set, we expect to see strongly clustered values of the success index above 0.98 (e.g. *LL:L*). Low values can highlight suspicious cases where the result mask may be inaccurate, for example because of motion artifact. Our results suggest that 0.96 is a suitable cutoff. The success index is particularly valuable when target cohorts are large and individual visual review of the brain masking results would be prohibitively time-consuming. A suitable strategy for a large set of target images might be to review those with the worst success index values, plus a random subset of the remainder.

Pincram is an accurate method for brain labelling on T1-weighted MR images of the adult human head that is distinguished by its robustness, versatility, and self-monitoring capability.

## Supporting Information

S1 File Raw mask quality assessment data from all experiments. Reference brain volume in mm^3^ (A.csv), overlap values for all pairings (B.csv), surface distance for all pairings (C.csv), success index for all relevant pairings (D.csv).
(ZIP)

## References

[1] Eritaia J, Wood SJ, Stuart GW, Bridle N, Dudgeon P, Maruff P, et al (2000) An optimized method for estimating intracranial volume from magnetic resonance images. Magnetic resonance in medicine 44: 973–977. 10.1002/1522-2594(200012)44:6<973::AID-MRM21>3.0.CO;2-H 11108637

[2] Freeborough PA, Fox NC, Kitney RI (1997) Interactive algorithms for the segmentation and quantitation of 3-D MRI brain scans. Computer Methods and Programs in Biomedicine 53: 15–25. 10.1016/S0169-2607(97)01803-8 9113464

[3] Lemieux L, Hammers A, Mackinnon T, Liu RS (2003) Automatic segmentation of the brain and intracranial cerebrospinal uid in T1-weighted volume MRI scans of the head, and its application to serial cerebral and intracranial volumetry. Magn Reson Med 49: 872–884. 10.1002/mrm.10436 12704770

[4] Smith SM (2002) Fast robust automated brain extraction. Human brain mapping 17: 143–155. 10.1002/hbm.10062 12391568 PMC6871816

[5] Ashburner J, Friston KJ (2005) Unified segmentation. NeuroImage 26: 839–851. 10.1016/j.neuroimage.2005.02.018 15955494

[6] Keihaninejad S, Heckemann RA, Fagiolo G, Symms MR, Hajnal JV, Hammers A. (2010) A robust method to estimate the intracranial volume across MRI field strengths (1.5T and 3T). NeuroImage 50: 1427–1437. 10.1016/j.neuroimage.2010.01.064 20114082 PMC2883144

[7] Leung KK, Barnes J, Modat M, Ridgway GR, Bartlett JW, Fox NC, et al (2011) Brain MAPS: an automated, accurate and robust brain extraction technique using a template library. NeuroImage 55: 1091–1108. 10.1016/j.neuroimage.2010.12.067 21195780 PMC3554789

[8] Eskildsen SF, Coupé P, Fonov V, Manjón JV, Leung KK, Guizard N, et al (2012) BEaST: brain extraction based on nonlocal segmentation technique. NeuroImage 59: 2362–2373. 10.1016/j.neuroimage.2011.09.012 21945694

[9] Manjón JV, Eskildsen SF, Coupé P, Romero JE, Collins DL, Robles M. (2014) Nonlocal intracranial cavity extraction. International journal of biomedical imaging 2014. 10.1155/2014/820205 PMC4195262 25328511

[10] Hammers A, Allom R, Koepp MJ, Free SL, Myers R, Lemieux L, et al (2003) Three-dimensional maximum probability atlas of the human brain, with particular reference to the temporal lobe. Hum Brain Mapp 19: 224–247. 10.1002/hbm.10123 12874777 PMC6871794

[11] Sled JG, Zijdenbos AP, Evans AC (1998) A nonparametric method for automatic correction of intensity nonuniformity in MRI data. IEEE transactions on medical imaging 17: 87–97. 10.1109/42.668698 9617910

[12] Landman BA, Warfield SK (2012) MICCAI 2012 Workshop on Multi-Atlas Labeling. In: Medical Image Computing and Computer Assisted Intervention Conference 2012: MICCAI 2012 Grand Challenge and Workshop on Multi-Atlas Labeling Challenge Results.

[13] Tustison NJ, Avants BB, Cook PA, Zheng Y, Egan A, Yushkevich PA, et al (2010) N4ITK: improved N3 bias correction. IEEE Transactions on Medical Imaging 29: 1310–1320. 10.1109/TMI.2010.2046908 20378467 PMC3071855

[14] Shattuck DW, Mirza M, Adisetiyo V, Hojatkashani C, Salamon G, Narr KL, et al (2008) Construction of a 3D probabilistic atlas of human cortical structures. NeuroImage 39: 1064–1080. 10.1016/j.neuroimage.2007.09.031 18037310 PMC2757616

[15] Rueckert D, Sonoda LI, Hayes C, Hill DL, Leach MO, Hawkes DJ. (1999) Nonrigid registration using free-form deformations: application to breast MR images. IEEE Transactions on Medical Imaging 18: 712–721. 10.1109/42.796284 10534053

[16] Heckemann RA, Keihaninejad S, Aljabar P, Rueckert D, Hajnal JV, Hammers A. (2010) Improving intersubject image registration using tissue-class information benefits robustness and accuracy of multi-atlas based anatomical segmentation. NeuroImage 51: 221–227. 10.1016/j.neuroimage.2010.01.072 20114079

[17] Studholme C, Hill DLG, Hawkes DJ (1999) An overlap invariant entropy measure of 3D medical image alignment. Pattern Recognition 32: 71–86. 10.1016/S0031-3203(98)00091-0

[18] Heckemann RA, Hajnal JV, Aljabar P, Rueckert D, Hammers A (2006) Automatic anatomical brain MRI segmentation combining label propagation and decision fusion. NeuroImage 33: 115–126. 10.1016/j.neuroimage.2006.05.061 16860573

[19] Shattuck DW, Prasad G, Mirza M, Narr KL, Toga AW (2009) Online resource for validation of brain segmentation methods. NeuroImage 45: 431–439. 10.1016/j.neuroimage.2008.10.066 19073267 PMC2757629

[20] Bajcsy R, Lieberson R, Reivich M (1983) A computerized system for the elastic matching of deformed radiographic images to idealized atlas images. Journal of Computer Assisted Tomography 7: 618–625. 10.1097/00004728-198308000-00008 6602820

[21] Aljabar P, Heckemann R, Hammers A, Hajnal JV, Rueckert D (2007) Classifier selection strategies for label fusion using large atlas databases. Medical image computing and computer-assisted intervention: MICCAI International Conference on Medical Image Computing and Computer-Assisted Intervention 10: 523–531. 10.1007/978-3-540-75757-3_64 18051099

[22] Langerak TR, van der Heide UA, Kotte AN, Viergever MA, van Vulpen M, Pluim JP. (2010) Label fusion in atlas-based segmentation using a selective and iterative method for performance level estimation (SIMPLE). IEEE Transactions on Medical Imaging 29: 2000–2008. 10.1109/TMI.2010.2057442 20667809

[23] Huang M, Yang W, Jiang J, Wu Y, Zhang Y, Chen W, eet al (2014) Brain extraction based on locally linear representation-based classification. NeuroImage 92: 322–339. 10.1016/j.neuroimage.2014.01.059 24525169

[24] Modat M, Ridgway GR, Taylor ZA, Lehmann M, Barnes J, Hawkes DJ, et al (2010) Fast free-form deformation using graphics processing units. Computer Methods and Programs in Biomedicine 98: 278–284. 10.1016/j.cmpb.2009.09.002 19818524

[25] Shi W, Jantsch M, Aljabar P, Pizarro L, Bai W, Wang H, et al (2013) Temporal sparse free-form deformations. Medical image analysis 17: 779–789. 10.1016/j.media.2013.04.010 23743085

[26] Gousias IS, Rueckert D, Heckemann RA, Dyet LE, Boardman JP, Edwards AD, et al (2008) Automatic segmentation of brain MRIs of 2-year-olds into 83 regions of interest. NeuroImage 40: 672–684. 10.1016/j.neuroimage.2007.11.034 18234511

[27] Gousias IS, Hammers A, Heckemann RA, Counsell SJ, Dyet LE, Boardman JP, et al. (2010) Atlas selection strategy for automatic segmentation of pediatric brain MRIs into 83 ROIs. In: Imaging Systems and Techniques (IST), 2010 IEEE International Conference on. IEEE, pp. 290–293.

[28] Gousias IS, Hammers A, Counsell SJ, Edwards A, Rueckert D (2012) Automatic segmentation of pediatric brain MRIs using a maximum probability pediatric atlas. In: Imaging Systems and Techniques (IST), 2012 IEEE International Conference on. IEEE, pp. 95–100.

[29] Gousias IS, Hammers A, Counsell SJ, Srinivasan L, Rutherford MA, Heckemann RA, et al (2013) Magnetic resonance imaging of the newborn brain: automatic segmentation of brain images into 50 anatomical regions. PLOS ONE 8. 10.1371/journal.pone.0059990 PMC3615077 23565180

