# Correction: Brain Extraction Using Label Propagation and Group Agreement: Pincram

**DOI:** 10.1371/journal.pone.0135746

**Published:** 2015-08-12

**Authors:** Rolf A. Heckemann, Christian Ledig, Katherine R. Gray, Paul Aljabar, Daniel Rueckert, Joseph V. Hajnal, Alexander Hammers

The images for Figs 1 and 2 are incorrectly switched. The image that appears as Fig 1 should be Fig 2, and the image that appears as Fig 2 should be Fig 1. The figure captions appear in the correct order.

**Fig 1:**
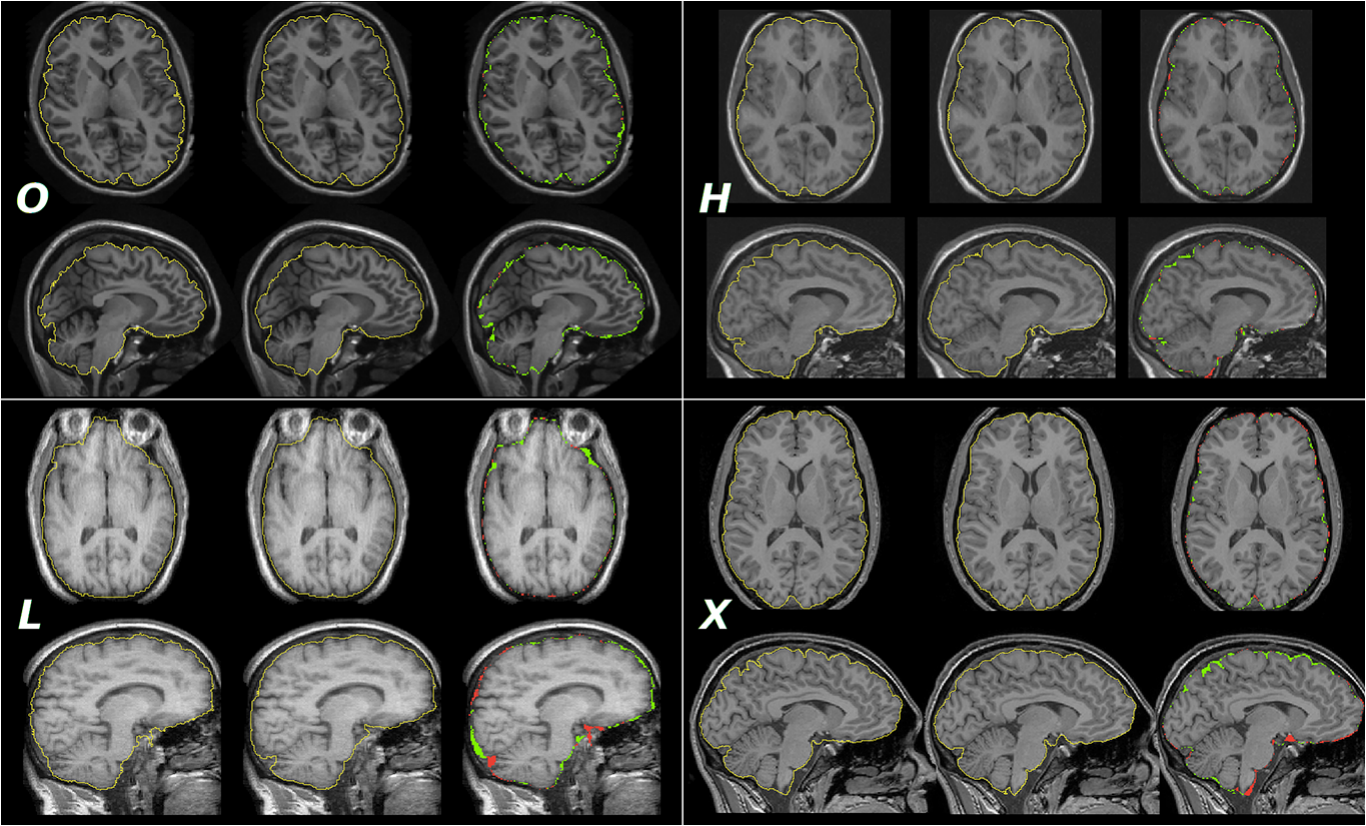
Sample images chosen randomly from each dataset. Images were visually centred at the level of the commissures approximately in the centre of the left thalamus to acquire a transverse (top rows) and a sagittal (bottom rows) slice. Left column, *O*, *H*, *L*: manual reference masks, *X*: generated mask (*OX*setup). Middle column, *O*, *H*, *L*, *X*: generated masks (*HX* setup in the case of X). Right column, *O*, *H*, *L*, *X*: discrepancies between the masks—green indicates overinclusion, red indicates underinclusion. Individual JCs were 0.9512 (*O*), 0.9704 (*H*), 0.9647 (*L*), and 0.9503 (*X*).

**Fig 2:**
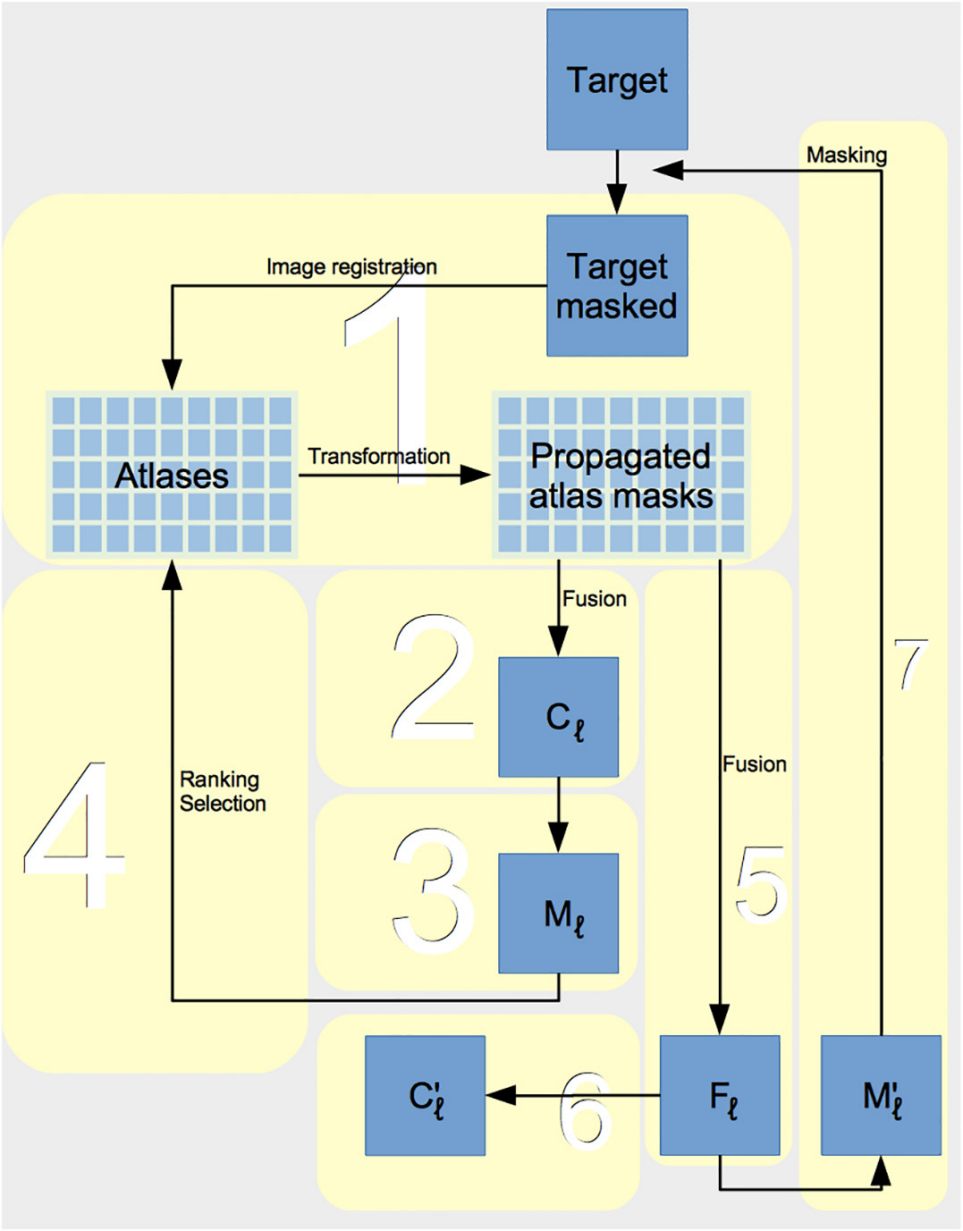
Overview diagram of pincram workflow. Step numbers in the text correspond to numbered boxes. *C*_*l*_: pre-selection fused mask;*M*_*l*_: tight margin (boundary neighborhood) mask; *F*_*l*_: fuzzy label summed from rank-selected subset; $C_{l}^{\prime }$: brain mask generated from *F*_*l*_ by thresholding and binarization; $M_{l}^{\prime }$ wide margin mask generated from *F*_*l*_ by thresholding and binarization

## References

[1] Heckemann RA, Ledig C, Gray KR, Aljabar P, Rueckert D, Hajnal JV, et al (2015) Brain Extraction Using Label Propagation and Group Agreement: Pincram. PLoS ONE 10(7): e0129211 doi: 10.1371/journal.pone.0129211 26161961 10.1371/journal.pone.0129211 PMC4498771

